# Expression of a Chloroplast-Targeted Cyanobacterial Flavodoxin in Tomato Plants Increases Harvest Index by Altering Plant Size and Productivity

**DOI:** 10.3389/fpls.2019.01432

**Published:** 2019-11-08

**Authors:** Martín L. Mayta, Rocío C. Arce, Matias D. Zurbriggen, Estela M. Valle, Mohammad-Reza Hajirezaei, María I. Zanor, Néstor Carrillo

**Affiliations:** ^1^Instituto de Biología Molecular y Celular de Rosario (IBR-UNR/CONICET), Facultad de Ciencias Bioquímicas y Farmacéuticas, Universidad Nacional de Rosario (UNR), Rosario, Argentina; ^2^Leibniz Institute of Plant Genetics and Crop Plant Research, Stadt Seeland, Germany

**Keywords:** tomato, flavodoxin, chloroplasts, transgenic plants, harvest index

## Abstract

Tomato is the most important horticultural crop worldwide. Domestication has led to the selection of highly fruited genotypes, and the harvest index (HI), defined as the ratio of fruit yield over total plant biomass, is usually employed as a biomarker of agronomic value. Improvement of HI might then result from increased fruit production and/or lower vegetative growth. Reduction in vegetative biomass has been accomplished in various plant species by expression of flavodoxin, an electron shuttle flavoprotein that interacts with redox-based pathways of chloroplasts including photosynthesis. However, the effect of this genetic intervention on the development of reproductive organs has not been investigated. We show herein that expression of a plastid-targeted cyanobacterial flavodoxin in tomato resulted in significant reduction of plant size affecting stems, leaves, and fruit. Decreased size correlated with smaller cells and was accompanied by higher pigment contents and photosynthetic activities per leaf cross-section. Flavodoxin accumulated in green fruit but declined with ripening. Significant increases in HI were observed in flavodoxin-expressing lines due to the production of higher fruit number per plant in smaller plants. Therefore, overall yields can be enhanced by increasing plant density in the field. Metabolic profiling of ripe red fruit showed that levels of sugars, organic acids, and amino acids were similar or higher in transgenic plants, indicating that there was no trade-off between increased HI and fruit metabolite contents in flavodoxin-expressing plants. Taken together, our results show that flavodoxin has the potential to improve major agronomic traits when introduced in tomato.

## Introduction

Harvest index (HI) is defined as the ratio of grain, fruit, or tuber yield to total plant biomass (Gur et al., 2010) and reflects the ability of a sink tissue to capitalize on the availability of photosynthates to increase the yield of harvestable product. As such, HI has been regarded as a reference parameter to evaluate the progress of breeding programs aimed at improving yield potential. Indeed, the so-called "green revolution" that took place in the middle of the twentieth century largely stemmed from major increases in HI resulting from the development of dwarf varieties of rice and wheat with diminished leaf biomass coupled to similar or higher grain yields (Khush, 2001). These dwarfing traits were found to result from mutations of genes involved in gibberellin synthesis and signaling (Hedden, 2003). Crossing of the mutant lines with high-yielding varieties led to new cultivars displaying a greater proportion of photoassimilates partitioned into the grain (Langridge, 2014).

Intensive research has been carried out to identify genes affecting vegetative and reproductive growth in a way that favors high HI. Association mapping in rice (Li et al., 2012) and rapeseed (Luo et al., 2015) indicated that HI is a complex multigenic trait affected by both environmental and genetic determinants. HI can be improved by favoring nutrient transport from leaves to harvestable organs and/or by decreasing vegetative growth (Luo et al., 2015). An early example of the latter approach was provided by tobacco plants over-expressing a phytochrome gene that exhibited impaired shade avoidance causing proximity-dependent dwarfing and higher HI (Robson et al., 1996). Although many quantitative trait loci associated with HI have been reported in various plant species, the specific genes and molecular mechanisms determining the magnitude of this index are largely unknown.

While a higher HI has a general relevance for crop yield enhancement, its agronomic value is particularly important in the case of species that are grown for industrial purposes (e.g. processing tomatoes) as a strategy to obtain smaller and more compact plants with equivalent or higher fruit production that are expected to render increased yields per planted surface (Gur et al., 2010).

Decreases in plant and leaf size have been observed in creeping bentgrass and *Arabidopsis* lines expressing a chloroplast-located flavodoxin (Fld; Li et al., 2017; Su et al., 2018). Fld is an electron shuttle flavoprotein found in cyanobacteria and some marine algae, which mediates essentially the same electron transfer reactions as the iron-sulfur protein ferredoxin (Fd; Pierella Karlusich et al., 2014). Fd transcript and protein levels are down-regulated by most environmental stresses (Pierella Karlusich et al., 2014, and references therein), and under such conditions Fld expression is induced to take over the activities of its functional counterpart and allow growth and reproduction of the microorganism in the adverse situation (Zurbriggen et al., 2008; Pierella Karlusich et al., 2014). Fld-encoding genes are absent from plant genomes (Pierella Karlusich et al., 2015), but introduction of a plastid-targeted Fld in transgenic plants resulted in increased tolerance to multiple sources of biotic and abiotic stress (Tognetti et al., 2006; Tognetti et al., 2007; Zurbriggen et al., 2008; Zurbriggen et al., 2009; Coba de la Peña et al., 2010; Li et al., 2017; Rossi et al., 2017).

In this study we transformed tomato plants with DNA sequences encoding a cyanobacterial Fld directed to chloroplasts (*Slpfld* lines, for *Solanum lycopersicum* **p**lastidic **Fld**) or the **c**ytosol (*Slcfld* lines), and evaluated vegetative and reproductive growth to determine if tomato HI could be increased by this genetic intervention. Mature-sized Fld was detected in leaves and fruit, but its levels declined with fruit ripening, in parallel with the general decline of total soluble protein. Lines expressing plastid-targeted Fld displayed a number of distinct phenotypic features compared to wild-type (WT) and *Slcfld* siblings, including smaller plants, leaves, and fruits; more flowers per inflorescence; increased fruit number; and higher HI. Biochemical analysis and metabolic profiling revealed that *Slpfld* fruit contained higher levels of soluble solids and similar or increased contents of sugars, amino acids, and organic acids relative to their WT counterparts. The results indicate that the chloroplast Fld approach constitutes a promising strategy to generate novel tomato lines displaying increased HI without affecting fruit metabolite contents.

## Materials and Methods

### Generation of Transgenic Tomato Lines

The *pfld*- and *cfld*-harboring pCAMBIA2200 plasmids (Tognetti et al., 2006; see **Supplementary Figure S1A**) were used to direct expression of Fld from *Anabaena* PCC7119 in the chloroplasts or the cytosol, respectively, of tomato plants (*S. lycopersicum* cv Moneymaker) by standard *Agrobacterium*-mediated procedures (Tauberger et al., 2000). A total of 22 *Slpfld* and 10 *Slcfld* transformants were obtained exhibiting detectable levels of Fld in leaf extracts. Typical examples are shown in **Supplementary Figure S2**. Homozygous lines were selected by evaluating resistance to 100 µg ml^−1^ kanamycin and by measuring Fld levels in the progeny of self-pollinated T2 transformants, using known amounts of purified recombinant Fld as reference (**Supplementary Figure S1B**). Leaf contents of the flavoprotein were analyzed by immunoblotting up to the T5 generation to ensure that the transgene was neither lost nor silenced during seed propagation.

Plants were germinated in soil and grown at 200 µmol photons m^−2^ s^−1^, 25°C, 40%/90% humidity with a 16/8-h light/dark photoperiod (growth chamber conditions) on randomly distributed 3-L pots. Watering was carried out daily to ﬁeld capacity until harvest at 120 days post-germination (dpg).

### Determination of Cell Size and Number

**Figure 1:**
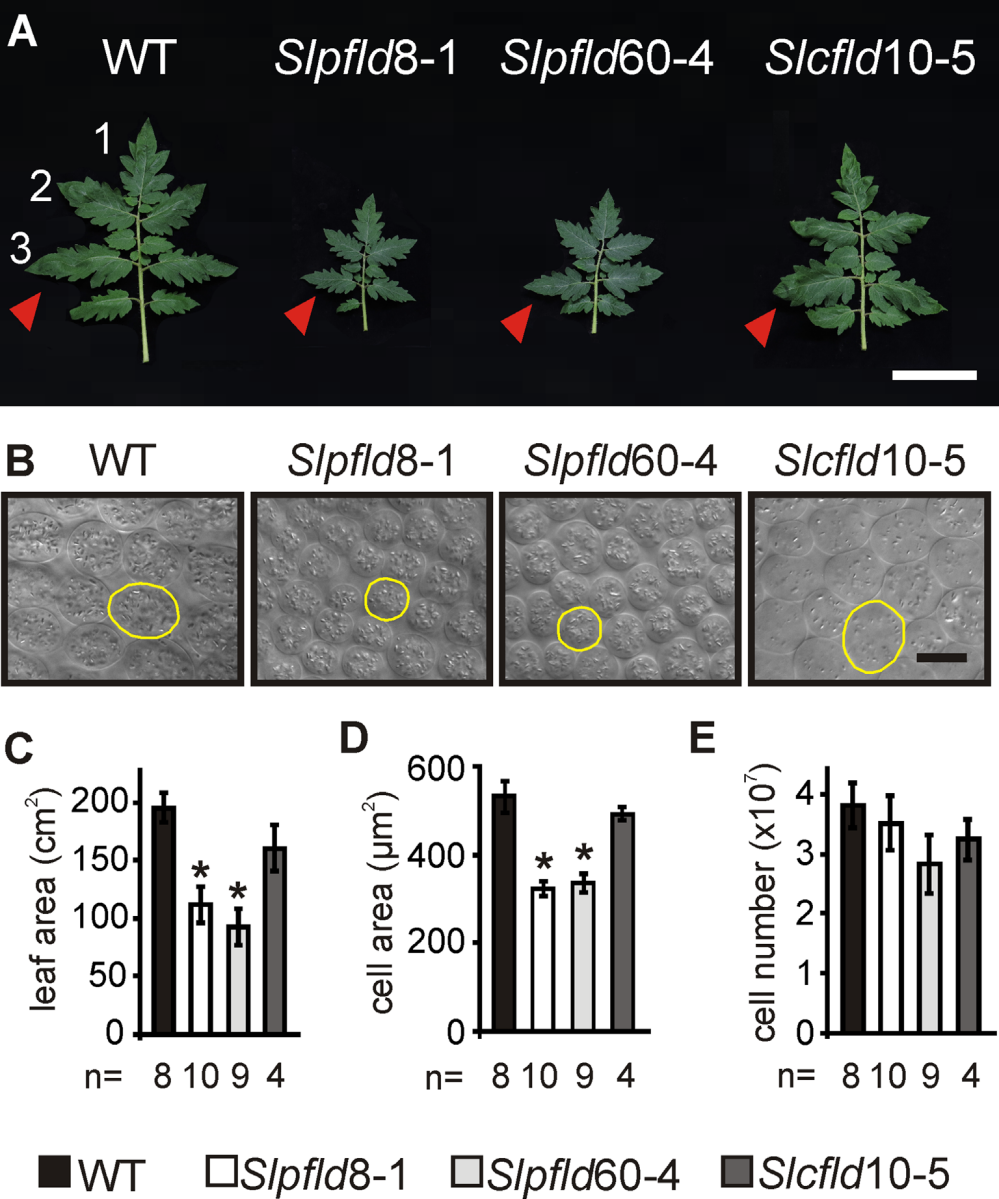
Flavodoxin expression in chloroplasts altered tomato leaf development. **(A)** Phenotypes of the fourth fully expanded leaves (counting from the bottom) of plants at 30 days post-germination. Bar = 10 cm. Arrowheads indicate the third leaflets used to extract tissue samples. **(B)** Representative micrographs from palisade parenchyma cells. Contour of typical cells are shown in yellow. Bar = 20 µm. **(C)** Leaf area was determined by image analysis. **(D)** Cell area and **(E)** cell number were calculated from clarified leaf tissue (see *Materials and Methods*). Data reported are means ± SEM of *n* biological replicates, as shown below each line. Asterisks indicate statistically significant differences (*P* < 0.05) determined using one-way ANOVA and Tukey's multiple comparison test.

Discs (0.5 cm in diameter) were punched from the interveinal region of the third leaflet from the fourth fully expanded leaf of several independent plants at 30 dpg (**Figure 1A**) and fixed in 96% (v/v) ethanol, followed by incubation in 85% (w/v) lactic acid for clearing. Four pictures of different regions in each disc were used to calculate cell area and at least 100 cells were counted. Cell number was estimated using leaf and cell areas. Image analysis was performed with ImageJ (Rasband, 1997–2008, http://rsb.info.nih.gov/ij/).

Fruit samples were analyzed at the breaker stage. Four thin (0.5–1 mm) transverse sections of one fruit from the first truss in six different plants per line (24 sections per line) were hand-cut from the equatorial section of the pericarp with a razor blade. The tissue was fixed in 10% (v/v) formaldehyde, 5% (v/v) acetic acid, and 52% (v/v) ethanol, vacuum-infiltrated twice for 15 min, and incubated overnight. The fixation solution was subsequently replaced by ethanol for 2 h, and pericarp tissues stored in 70% (v/v) ethanol until processing. For cell size studies, samples were stained by incubation with 0.5% (w/v) toluidine blue in 0.1% (w/v) Na_2_CO_3_ for 30 s. Samples were rinsed with water to prevent staining of the internal cell layers and mounted in 30% (v/v) glycerol. Stained sections were photographed using a camera attached to a dissecting microscope (Leica Microsystems, Switzerland). Cell layers were counted four times (technical replicates) in each fruit section from the exocarp to endocarp avoiding the vascular bundles, as described by Mu et al. (2017). Cell size was calculated using ImageJ (Rasband, 1997–2008, http://rsb.info.nih.gov/ij/).

### Phenotyping

For seed viability and germination analysis, seeds were cultured under growth chamber conditions on 0.8% (w/v) agar plates containing half-strength Murashige-Skoog basal salts (Sigma). Germination was recorded from the day the radicle broke through the seed coat. Groups of 30 seeds were used for each germination test, and the assay was repeated four times (independent experiments). Time to leaf emergence was determined for the emergence of the first and second node in 12 to 13 plants of each line germinated at the same time and grown in soil. Inflorescence and flower counting and tagging were performed every 2 days. Fruit ripening stages were determined by epicarp color change and by pressing it gently. They were classified as immature green (∼10 days post-anthesis), mature green (∼50 days post-anthesis), breaker, and ripe red, when they changed to a dark red color and soft texture. For biochemical and physiological measurements, fruit of equivalent developmental stages were employed, irrespective of their days from anthesis. To determine yield and HI, ripe red fruit were collected daily up to 120 dpg. At this stage, all remaining fruit were harvested irrespective of their ripening stage, and used for calculations. Dry weight was recorded after 2 weeks of incubation at 65°C. Vegetative weight (leaves and stems) was determined after fruit harvest, and HI was calculated as the ratio between total fruit yield and total above-ground biomass (fruit plus vegetative). Data shown were averaged from three independent experiments carried out during a 2-year period.

### Leaf Pigment Contents and Photosynthetic Measurements

Chlorophyll (*Chl*) and carotenoid levels were determined spectrophotometrically after extraction with 96% (v/v) ethanol (Lichtenthaler, 1987). Chlorophyll fluorescence measurements were performed using a MultispeQ-Beta device controlled by the PhotosynQ platform software (Kuhlgert et al., 2016). Measurements were performed on leaves from the fifth node in two fully expanded leaflets using six independent plants per line at 45 dpg.

### Metabolite Quantifications

For determination of fruit metabolites, two ripe red fruit were sampled from the first truss of at least three individual plants (biological replicates) from each genotype. Metabolite profiling was performed by proton nuclear magnetic resonance (^1^H-NMR) spectroscopy according to published procedures (Sorrequieta et al., 2013; López et al., 2015). Briefly, pericarp tissue of ripe red fruit was obtained by removing the epicarp, locule tissues, and seeds, immediately frozen in liquid nitrogen and stored at −80°C until analysis of the primary metabolite composition by ^1^H-NMR. Frozen samples were ground in liquid nitrogen using a Retsch MM400 mixer mill until obtaining a homogeneous and fine powder, which was rapidly dissolved in 0.3 ml of 1 M cold sodium phosphate buffer (pH 7.4) prepared in D_2_O to obtain a mixture containing about 30% by weight of D_2_O. The mixture was centrifuged at 20,000*g* for 15 min at 4°C and the supernatant filtered to remove any insoluble material. Internal standard [1 mM TSP: 3-(trimethylsilyl) propionic-2,2,3,3-d4 acid] was added to the resulting transparent soluble fraction, and the solution was subjected to spectral analysis at 600.13 MHz on a Bruker Avance II spectrometer. Proton spectra were acquired at 298 K by adding 512 transients of 32 K data points with a relaxation delay of 5 s. A 1D-NOESY pulse sequence was utilized to remove the water signal. The 90° flip angle pulse was always ∼10 µs. Proton spectra were referenced to the TSP signal (δ = 0 ppm), and their intensities were scaled to that of TSP. Spectral assignment and identification of specific metabolites was established by fitting the reference proton nuclear magnetic spectroscopy spectra of several compounds using the software Mixtures, developed *ad hoc* as an alternative to commercial programs (Abriata, 2012). Further confirmation of the assignments for some metabolites was obtained by acquisition of new spectra after addition of authentic standards.

### Analytical Procedures

Fld levels in the various tissues were estimated by sodium dodecyl sulfate–polyacrylamide gel electrophoresis (SDS-PAGE) and immunoblotting (Tognetti et al., 2006). Total protein extracts were prepared by grinding 100 mg tissue powder in 200 µL protein extraction buffer [0.2 M Tris-HCl, pH 6.8, 3 M urea, 1% (v/v) glycerol, 8% (w/v) SDS, 0.5 mM dithiothreitol, 5% (v/v) β-mercaptoethanol]. The composition of this buffer allows a more efficient protein extraction (Steiner et al., 2016). Samples were vortexed, incubated for 20 min at 80°C and centrifuged at 13,000*g* for 15 min. Supernatants were subjected to SDS-PAGE on 15% polyacrylamide gels and transferred to nitrocellulose membranes. Gel loading was carried out on the basis of fresh weight (FW) to avoid major changes in protein patterns and levels among the various tissues (see below). Membranes were washed three times for 15 min each with 5% (w/v) skim milk in 0.01% (v/v) Tween phosphate-buffered saline (TPBS; 8 mM Na_2_HPO_4_, 2 mM KH_2_PO_4_ pH 7.4, 137 mM NaCl, 2.7 mM KCl) and incubated for 1 h with polyclonal antibodies raised in rabbits against *Anabaena* Fld (diluted 1:300 in TPBS). Following washing with TPBS (three times × 15 min), membranes were incubated with rabbit anti-IgG immunoglobulins conjugated to alkaline phosphatase (Bio-Rad), in a 1:3,000 ratio in TPBS. After washing with TPBS (three times × 15 min), membranes were finally incubated in phosphatase solution (100 mM Tris-HCl pH 9.5, 100 mM NaCl, 5 mM MgCl_2_) supplemented with 0.01% (w/v) 5-bromo-4-chloro-3-indolyl phosphate and 0.01% (w/v) nitroblue tetrazolium until color development.

Total protein concentrations were measured in cleared leaf and fruit extracts as described by Simonian (2002), using bovine serum albumin as standard. The concentration of puriﬁed recombinant fld was determined by the absorption of bound ﬂavin mononucleotide (ε_454_ = 8.8 mM^−1^ cm^−1^).

Concentrations of total soluble solids were measured in duplicate as described by Zanor et al. (2009) using a portable MA871 Digital Brix refractometer in a random sample of six fruit per line. Results were expressed in Brix degrees.

### Statistical Analyses

Data were analyzed using one-way ANOVA and multiple range tests as specified in each experiment. Signiﬁcant differences refer to statistical signiﬁcance at *p* < 0.05.

## Results

### Expression of a Plastid-Targeted Fld Decreases Tomato Plant Size

To generate tomato plants expressing a plastid-targeted Fld (*Slpfld* lines), the coding region of the *Anabaena* PCC7119 *fld* gene was fused in-frame to the 3' end of a DNA sequence encoding the chloroplast transit peptide of pea Fd-NADP^+^ reductase (Tognetti et al., 2006; see *Materials and Methods*). The fused gene was cloned under the control of the constitutive cauliﬂower mosaic virus (CaMV) 35S promoter (**Supplementary Figure S1A**). A construct lacking the transit peptide sequence was also prepared to generate plants in which the expressed flavoprotein accumulated in the cytosol (*Slcfld* lines; **Supplementary Figure S1A**). The presence of Fld in foliar tissue was evaluated by SDS-PAGE and immunoblot analysis. Most of the flavoprotein was recovered as mature-sized peptides in *Slpfld* lines (**Supplementary Figure S1B**), indicating that it was imported by chloroplasts as already shown for Fld-expressing tobacco plants (Tognetti et al., 2006; Ceccoli et al., 2012). Homozygous lines were selected by segregation analysis and confirmed by proportional increases in leaf Fld contents. Lines *Slpﬂd*8-1, *Slpﬂd*60-4, and *Slcfld*10-5, belonging to the T5 generation and displaying high levels of Fld in chloroplasts or the cytosol, were used for phenotypic characterization.

Fld expression in *Slpfld* and *Slcfld* lines did not affect seed viability, germination rates, and time to leaf setting (**Supplementary Figure S3**), indicating that there was no retardation of vegetative development in the transformants. However, *Slpﬂd* plants exhibited smaller leaves and leaflets and shorter rachis compared to WT and *Slcﬂd* siblings (**Figures 1A, C**; **Supplementary Figure S4**; **Supplementary Table S1**), in agreement with the leaf phenotypes displayed by chloroplast Fld-expressing creeping bentgrass (Li et al., 2017) and *Arabidopsis* (Su et al., 2018). The number of leaflets per compound leaf and their overall architecture were instead unchanged (**Figure 1A**). Decreases in internodal distances of ∼30% accounted for stem shortening in *Slpﬂd* plants relative to WT counterparts (**Supplementary Table S1**). Overall size reduction was accompanied by significant decreases in FW and dry weight of the aerial parts (**Supplementary Table S1**). Stem diameter and relative water contents were not affected by the presence of the flavoprotein (**Supplementary Table S1**).

**Figure 2:**
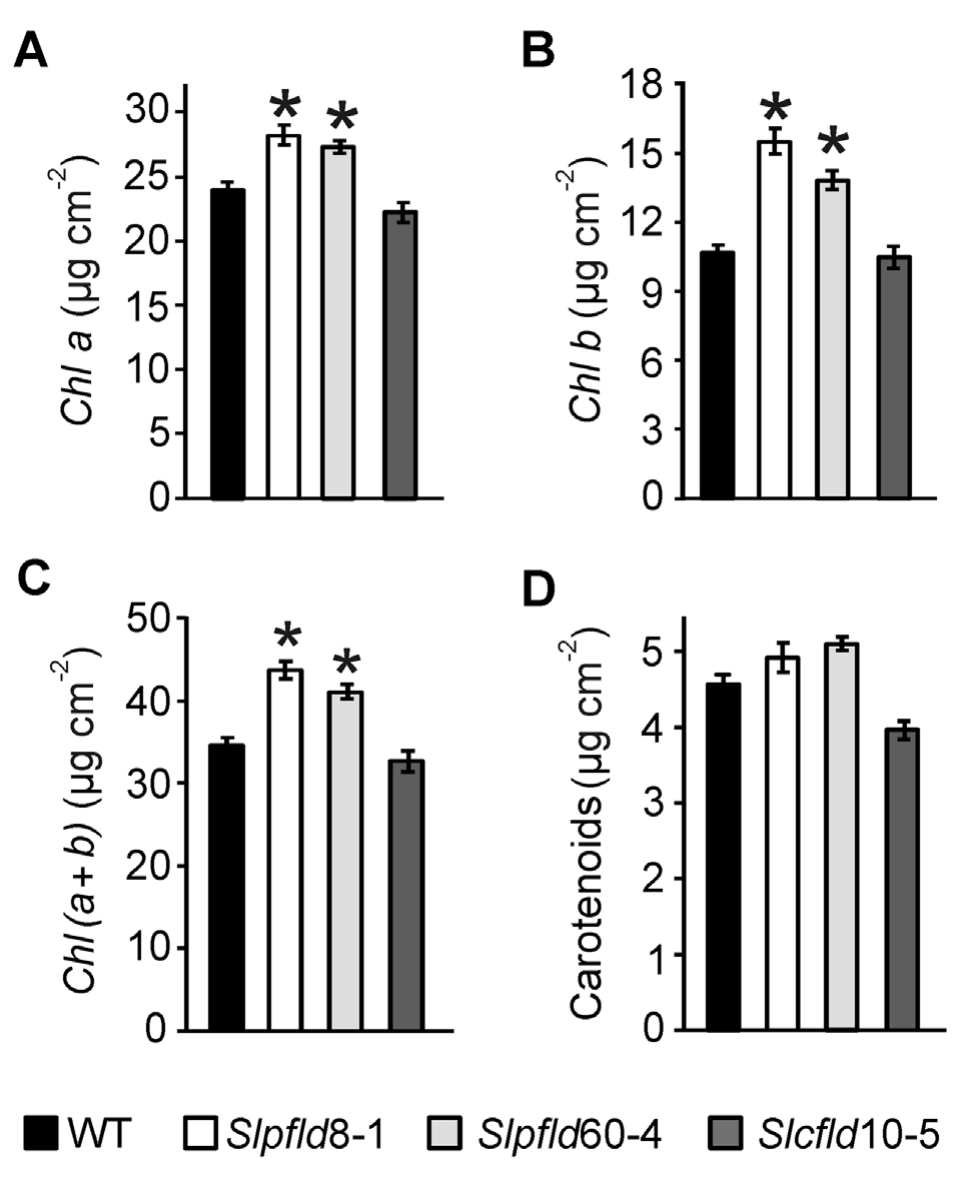
Tomato leaves expressing flavodoxin in chloroplasts displayed higher chlorophyll content. **(A)** Chlorophyll (*Chl*) *a*, **(B)** *Chl b*, **(C)** *Chl (a+b)*, and **(D)** carotenoids were determined in the third leaflet of the fourth fully expanded leaf of plants at 64 days post-germination. Data shown are means ± SEM of six biological replicates. Statistical differences between wild-type and transgenic lines are indicated by asterisks and were determined using one-way ANOVA and Tukey's Multiple Comparison Test (*P* < 0.05).

Leaf area reduction in *Slpﬂd* plants resulted from a decrease in mesophyll cell size, without significant changes in cell number (**Figures 1B, D, E**). Epidermal cells were also significantly smaller (**Supplementary Figure S5**). As in tobacco (Tognetti et al., 2006; Ceccoli et al., 2012; Mayta et al., 2018), *Slpﬂd* plants contained higher pigment contents per leaf area (**Figure 2**). *Chl a* and *Chl b* levels were 17% and 39% higher than those determined in WT and *Slcfld* leaves (**Figures 2A–C**). Marginal increases in carotenoids were also observed in a number of experiments, albeit without statistical significance (**Figure 2D**).

**Figure 3:**
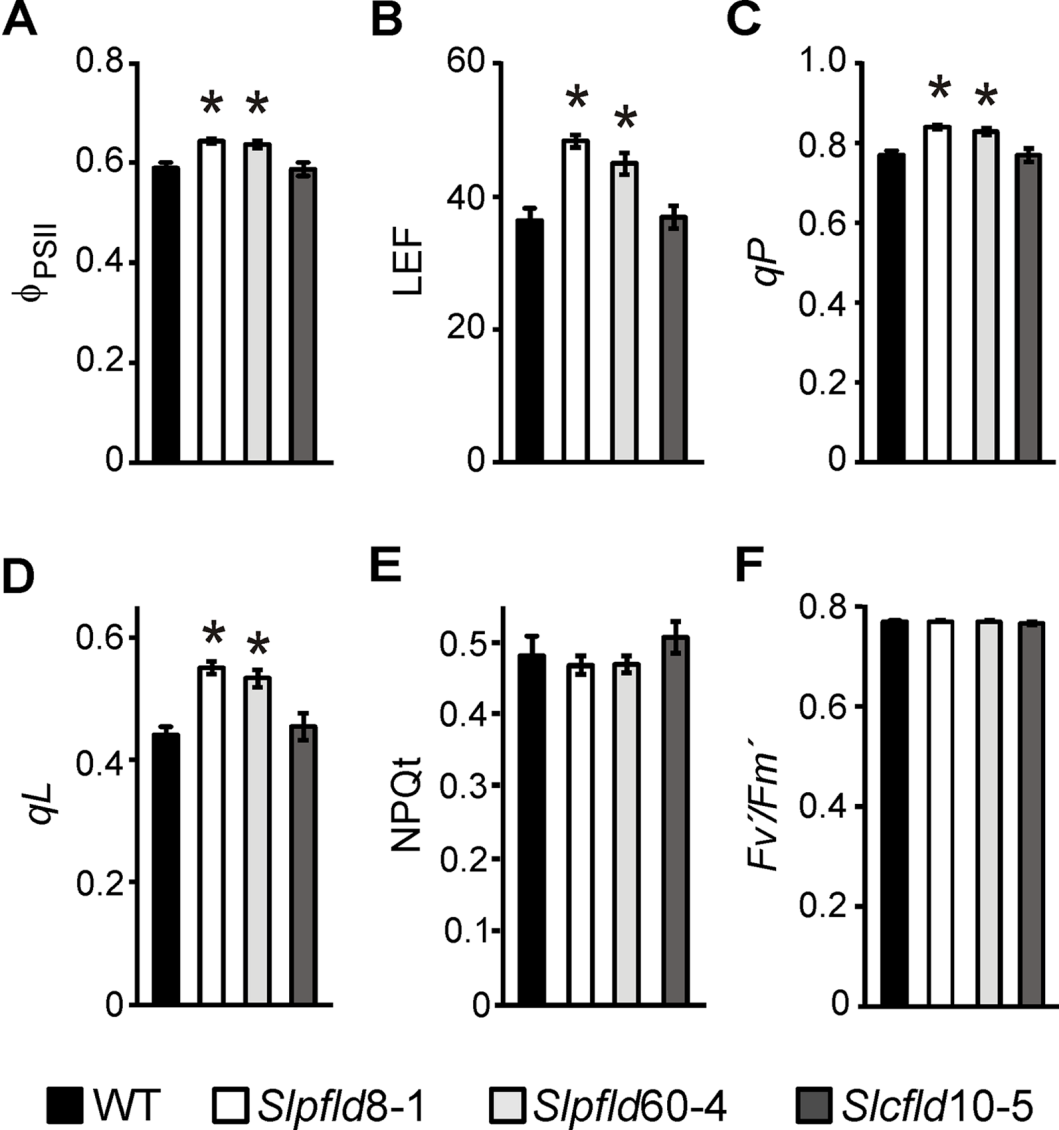
Tomato leaves expressing chloroplast flavodoxin exhibited higher photosynthetic activities. Measurements of quantum yield of photosystem (PS) II (ϕ_PSII_, **A**), rate of linear electron transport (LEF, **B**), photochemical quenching (*qP*, **C**), fraction of open PSII reaction centers (*qL*, **D**), non-photochemical quenching (NPQt, **E**), and PSII integrity (*F* *_v_* *′*/*F* *_m_* *′*, **F**) were carried out in the third leaflet of the fourth fully expanded leaf of plants at 45 days post-germination. They correspond to the means ± SEM of six biological samples with six technical replicates each. Statistical differences between wild-type and transgenic lines are indicated by asterisks and were determined using one-way ANOVA and Tukey's multiple comparison test (*P* < 0.05).

Higher *Chl* contents in *Slpfld* plants were reflected at the level of photosynthetic activities. The quantum yield of photosystem (PS) II (Ф_PSII_) per leaf cross-section, which provides an estimation of the electron ﬂow through PSII (Baker, 2008), and the rate of linear electron flow (LEF), were significantly enhanced in *Slpfld* plants at 45 dpg compared to the WT and *Slcfld* genotypes (**Figures 3A, B**). Other relevant photosynthetic parameters such as the coefficient of photochemical quenching *qP* and the fraction of open PSII reaction centers *qL* were also higher in *Slpfld* transformants (**Figures 3C, D**), whereas the magnitude of non-photochemical quenching (NPQt), which reflects the ability of the photosynthetic electron transport chain (PETC) to dissipate light energy into various processes (Baker, 2008), did not vary significantly among genotypes (**Figure 3E**). Similarly, the *F* *_v_* *′*/*F* *_m_* *′* parameter, which is regarded as a measure of PSII integrity, was not affected by Fld expression in chloroplasts or cytosol (**Figure 3F**).

### Plants Expressing Chloroplast-Located Fld Produce a Higher Number of Smaller Fruit

**Figure 4:**
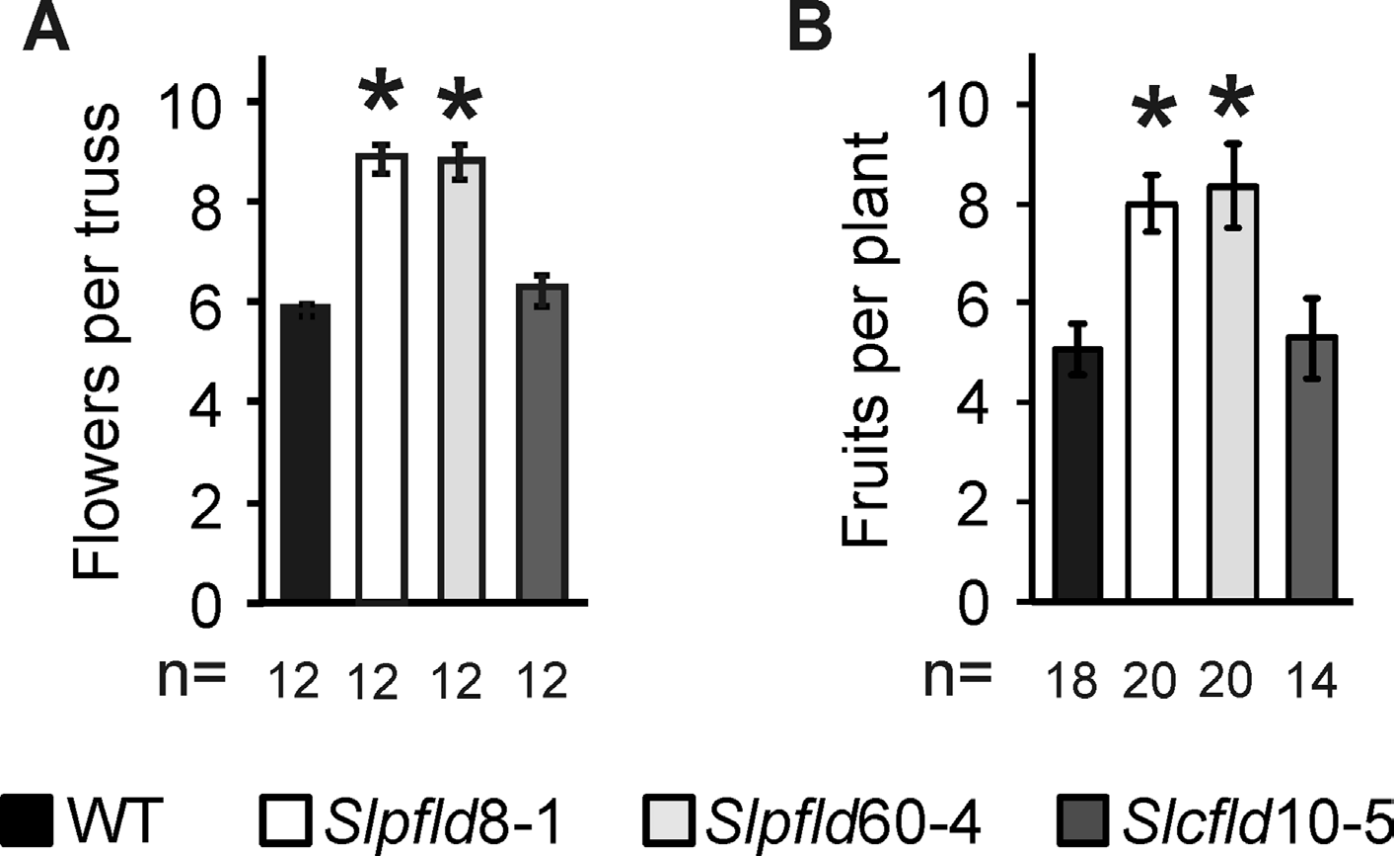
Flavodoxin expression in chloroplasts enhanced flower number and fruit production. **(A)** Flowers were counted in the three to five initial trusses from each plant. **(B)** Total fruit numbers per plant included fully ripe, breaker, and green fruit collected up to 120 days post-germination. Data are means ± SEM of *n* biological replicates, with *n* indicated below each line. Statistically significant differences are shown by asterisks and were determined using one-way ANOVA and Tukey's multiple comparison test (*P* < 0.05).

Flowering time was retarded in plants expressing a chloroplast Fld relative to WT and *Slcfld* siblings, as indicated by a delay of 10 to 12 days to the blossoming of the first flower (**Supplementary Figures S6A, B**). As a result, *Slpfld* plants had normally developed two more leaves on average at the time of flower setting (**Supplementary Figure S6C**). The number of tomato inflorescences and their architecture depend on the cultivar and are dramatically affected by environmental factors (Gur et al., 2010). Tomato trusses typically bear five to six flowers organized in a zigzag branch (Lippman et al., 2008). While the total inflorescences produced by *Slpﬂd* plants did not differ from the other two lines, they developed ∼50% more flowers per truss (**Figure 4A**; **Supplementary Table S2**). Delay in flowering might cause additional branching of the inflorescence, as previously reported (Lippman et al., 2008; Park et al., 2012), but the mechanism by which chloroplast-targeted Fld exerts this effect is at present unknown.

The presence of extra flowers resulted in more prolific fruit production per plant (**Figure 4B**; **Supplementary Table S2**). Interestingly, the delay in flowering was partially compensated by accelerated ripening in *Slpfld* plants, with both color break and fruit ripening occurring 6 to 7 days (counting from anthesis) earlier than WT and *Slcfld* siblings (**Supplementary Table S2**). To estimate yield and HI, fruits were collected as they ripened up to 120 dpg. At this time, all tomatoes remaining in the plant were harvested irrespective of their maturation stage.

Fld activity in chloroplasts depends on interaction with endogenous redox partners, most conspicuously the PETC (Pierella Karlusich et al., 2014). The redox chemistry of chloroplasts closely resembles that of the phototrophic microorganisms in which the flavoprotein is normally found, but Fld expression levels and possible interaction(s) in non-photosynthetic plastids such as those present in red fruit remain unknown. It should be borne in mind, however, that fruits stay green during a large part of their development, before the conversion of chloroplasts into chromoplasts (Marano and Carrillo, 1991; Marano et al., 1993; Muñoz and Munné-Bosch, 2018).

**Figure 5:**
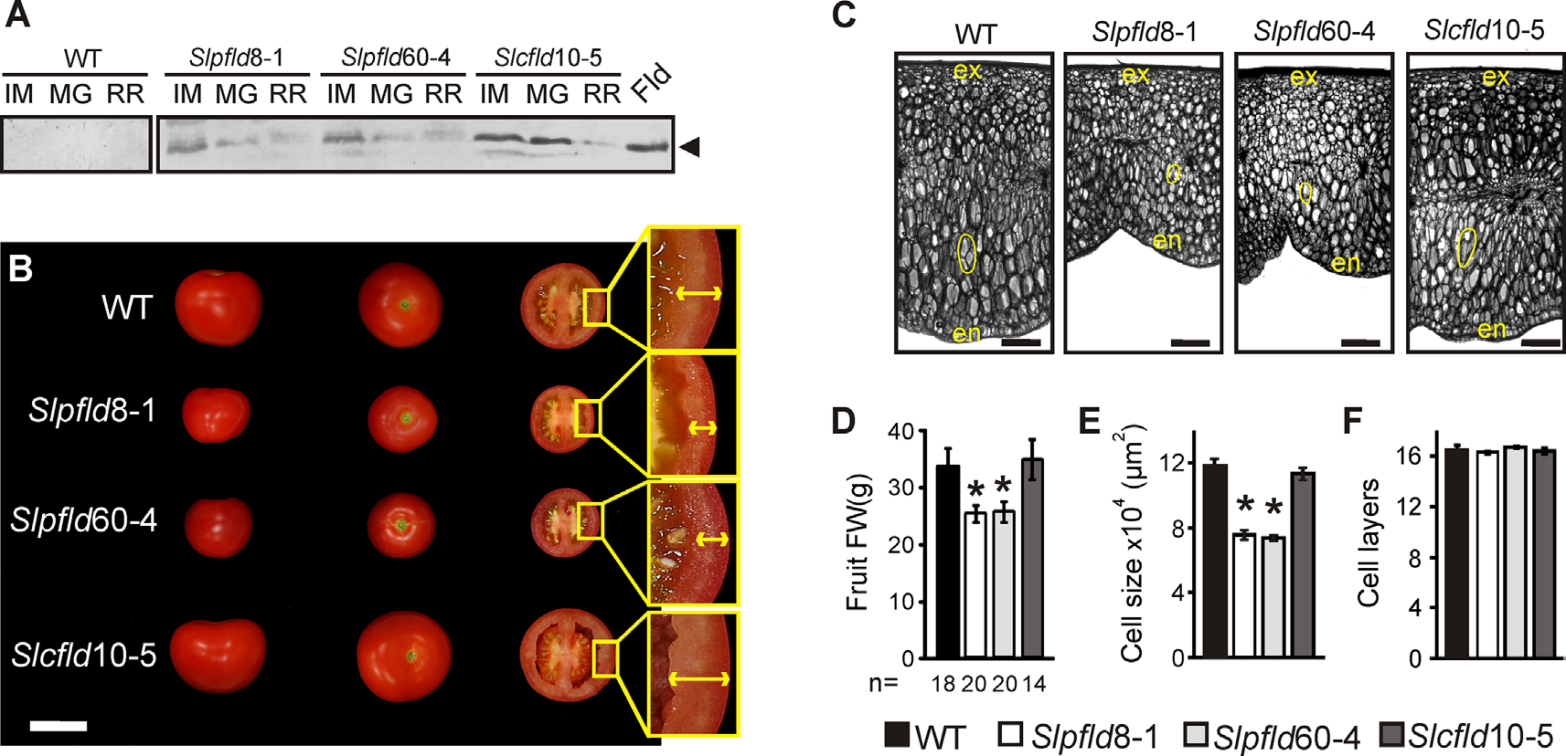
Plastid-located flavodoxin (Fld) affected tomato fruit development. **(A)** Fld expression at different fruit stages: immature green (IM), mature green (MG), and ripe red (RR). Cleared extracts corresponding to 5 mg FW were loaded in each lane, resolved by 15% sodium dodecyl sulfate–polyacrylamide gel electrophoresis and analyzed by immunoblot using Fld antisera, as described in *Materials and Methods*. The two membranes were assayed together and over-reacted to reveal unspecific staining. Purified Fld (0.8 pmol) is shown in the extreme right. MW: molecular weight marker. **(B)** Phenotypes of representative fruits from each line. Bar = 5 cm. Insets show pericarps delineated by arrows. **(C)** Pericarp sections from representative breaker fruits stained with toluidine blue. The inner epidermis is denoted as "en" and the outer epidermis as "ex." Typical cells are contoured in yellow to illustrate size differences. Bar = 1 mm. **(D)** Average fruit weight, *n* indicates the number of fruit assayed. Cell size **(E)** and the number of cell layers of the pericarp **(F)** were calculated from six biological replicates using sections as those depicted in panel (C). Data presented correspond to means ± SEM. Statistically significant differences are indicated by asterisks and were determined using one-way ANOVA and Tukey's multiple comparison test (*P* < 0.05).

Immunoblot analyses were used to estimate Fld accumulation in fruit tissues. Since ripening proceeded at a different pace in the various genotypes (**Supplementary Table S2**), fruit at equivalent ripening stages (different days from anthesis) were used by employing the classification described in *Materials and Methods*. Levels of total soluble protein were lower in immature green fruits compared to leaves, and declined further in all lines as ripening progressed (**Supplementary Figures S7** and **S8**). Gels for Fld detection were therefore loaded on the basis of FW, considering that unlike protein levels, the fraction of dry matter did not change significantly between tomato ripening stages (Matsuda and Kubota, 2010; Radzevičius et al., 2016). **Figure 5A** shows that mature-sized Fld was expressed in the green pericarp of immature *Slpfld* and *Slcfld* fruit. At more advanced ripening stages, chloroplast Fld levels declined together with total protein, to become barely detectable in ripe red fruit (**Figure 5A**; **Supplementary Figure S8B**). Contents of the cytosol-targeted flavoprotein were instead maintained up to the mature green stage (**Supplementary Figure S8B**), resulting in a relative Fld enrichment within total soluble proteins (**Supplementary Figure S8C**). Full ripening led to down-regulation of cytosolic Fld levels to those of *Slpfld* plants (**Supplementary Figure S8**).

Fld is therefore most likely functional in the green developmental stages, and fruit of *Slpfld* plants were smaller on average than those of the WT (**Figures 5B, D**), resembling the leaf phenotype. As in leaves, this effect was caused by a reduction in cell size (**Figures 5C, E**), while the number of cell layers in a transverse section of the pericarp was similar between WT and *Slpfld* fruit (**Figure 5F**), resulting in transformants with a thinner pericarp (**Figure 5B**).

**Figure 6:**
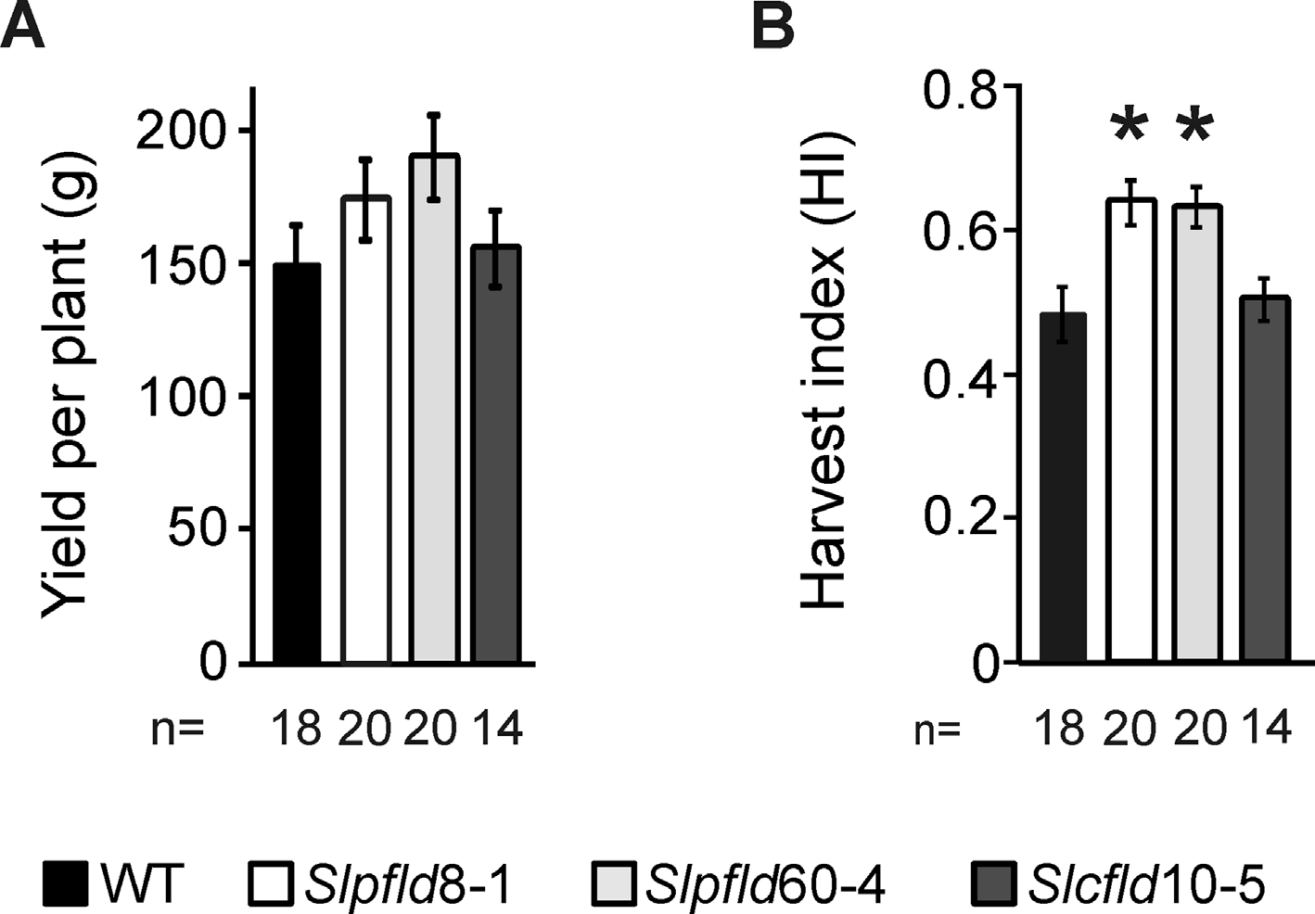
Chloroplast flavodoxin expression increased fruit harvest index. **(A)** Yield was estimated as the sum of all fruit harvested up to 120 days post-germination regardless of the ripening stage. **(B)** Harvest index was calculated as described in *Materials and Methods*. Data reported are means ± SEM of *n* biological replicates, as shown below each line. Statistically significant differences are indicated by asterisks and were determined using one-way ANOVA and Tukey's multiple comparison test (*P* < 0.05).

The compromise between higher fruit number and smaller fruit size and weight resolved in moderately increased fruit yields in *Slpfld* plants relative to those of WT siblings, but the small differences failed to show statistical significance (**Figure 6A**). In turn, the combination of similar total fruit weight per plant with lower vegetative biomass (**Supplementary Table S1**) led to a ∼30% increase in the HI of *Slpfld* lines compared to WT and *Slcfld* genotypes (**Figure 6B**).

### Metabolite Profiling of Ripe Red Tomato Fruit

**Figure 7:**
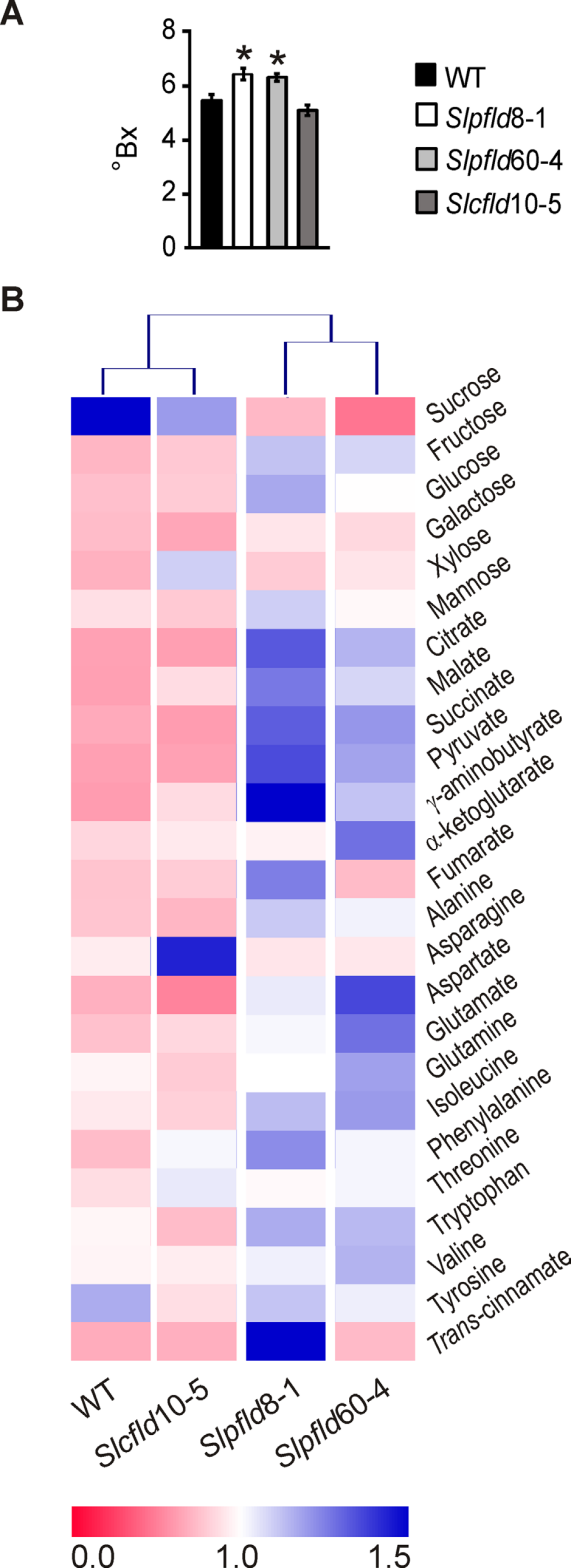
Flavodoxin expression in plastids led to higher soluble solids and metabolite contents in ripe red tomato fruits. **(A)** Brix Index (°Bx) was determined in at least two fruit from six different plants per genotype (12 fruit per line) using a portable refractometer as described in *Materials and Methods*. Data reported are means ± SEM. Statistically significant differences are indicated by asterisks and were determined using one-way ANOVA and Tukey's multiple comparison test (*P* < 0.05). **(B)** Heat map of metabolites assayed in the different lines. Values for each compound were normalized with respect to the mean from all lines, and rectangles represent the normalized amount of each metabolite using a false-color scale. Quantitative metabolite profiling was performed in two ripe red fruit of at least three individual plants by ^1^H-nuclear magnetic resonance spectroscopy as described in *Materials and Methods*. Heat map was designed with Multiple experiment Viewer (MeV).

While chloroplast Fld increased HI and fruit production, this improvement could be detrimental to fruit quality and nutrient contents. Determination of soluble solids measured in Brix degrees provides a fast and reliable indicator of fruit quality. As shown in **Figure 7A**, *Slpfld* plants displayed a moderate but statistically significant increase in pericarp soluble solids, indicating that reduction of photosynthetically active tissue in these lines was not translated into lower sugar accumulation in the sink organ. Indeed, fruit dry weight and relative water content were similar in all lines (**Supplementary Table S2**).

Quantitative metabolite profiling was performed in ripe red fruit of all lines to further assess the effects of Fld expression on nutrient contents of the marketable product (**Figure 7B**). With the conspicuous exception of sucrose, which declined in *Slpfld* fruit relative to WT siblings, soluble sugars displayed similar (galactose, xylose, mannose) or increased (glucose, fructose) levels in both lines expressing chloroplast Fld (**Supplementary Table S3**). Intermediates of central metabolism such as pyruvate, citrate, succinate, malate, and γ-aminobutyrate also exhibited higher contents in *Slpfld* fruit, whereas fumarate and α-ketoglutarate levels did not show significant differences among genotypes (**Supplementary Table S3**). Similarly, accumulation of the 11 proteinogenic amino acids measured was not affected by Fld presence (**Figure 7B**; **Supplementary Table S3**). In line with the moderate differences in Fld accumulation (**Figure 5A**; **Supplementary Figure S1B**), the two *Slpfld* lines showed a similar trend, without statistically significant differences, for most metabolites, whereas fruit from *Slcfld* plants in which Fld accumulated in the cytosol displayed metabolic profiles that more closely resemble those of their WT counterparts (**Figure 7B**; **Supplementary Table S3**). The only conspicuous exception was *trans*-cinnamic acid, which contents showed a major increase only in *Slpfld*8-1 plants, but remained at WT levels in *Slpfld*60-4 siblings (**Supplementary Table S3**).

The collected results indicate that the increased HI of plants expressing a chloroplast-located Fld was accompanied by similar or higher contents of sugars and other metabolites in ripe fruit, underscoring the potential value of the introduced trait.

## Discussion

Tomato domestication has been conducted over the centuries to select varieties with increased fruit weight and number. These traits are genetically controlled through a few *loci* associated with carpel anatomy and cell proliferation (Chakrabarti et al., 2013), but phytohormones and environmental and/or metabolic conditions, most conspicuously photosynthetic activity in source tissues, also affect fruit development (Ariizumi et al., 2013). Searching for traits that can reduce plant size while increasing HI is a most relevant objective and accordingly, limited vegetative growth is generally a desirable trait in crops (Gur et al., 2010).

Expression of a cyanobacterial Fld targeted to chloroplasts of various species led to significant decreases in vegetative growth, but the effects of this intervention on the development of reproductive organs were not reported (Li et al., 2017; Su et al., 2018). We addressed herein this question by expressing a plastid-targeted Fld in a commercial variety of tomato, and found a similar reduction in plant size (**Figure 1**; **Supplementary Figures S3** and **S4**; **Supplementary Table S1**). Decreased leaf area correlated with lower cell size (**Figure 1**), and was accompanied by higher *Chl* contents and photosynthetic activities per leaf cross-section (**Figures 2** and **3**). Fld was expressed in green fruit but its levels decreased with the progress of fruit ripening (**Figure 5A**; **Supplementary Figure S8**). Since expression of the flavoprotein was driven by a constitutive promoter (**Supplementary Figure S1A**), down-regulation of Fld contents with fruit ripening presumably involved post-transcriptional and/or post-translational mechanisms, including changes in the rates of protein synthesis and/or degradation (responsible for total protein decrease) and in the case of plastid-targeted Fld, alterations of import capacity during the transition leading to chromoplast formation (Sadali et al., 2019).

Chloroplast Fld exerted opposite effects on flowering time and fruit ripening (**Supplementary Figure S6**; **Supplementary Table S2**). Flowering time and the subsequent processes of flower patterning and fruit development are regulated by a different suite of genes, although common players do exist (Zhu and Helliwell, 2011). Association of flowering time to redox poise has been reported (Shim and Imazumi, 2015), and it is tempting to speculate that chloroplast Fld could affect this balance through its electron shuttling activity, but further research will be required to address this issue.

Plants accumulating Fld in plastids produced a higher number of smaller fruit, leading to increased HI (**Figures 5** and **6**; **Supplementary Table S2**) without detrimental effects on metabolite contents (**Figure 7**; **Supplementary Table S3**). Actually, ripe red fruit of *Slpfld* lines contained increased levels of soluble sugars (glucose and fructose) and organic acids (mainly citrate and malate). The higher hexose contents could indicate an increase of invertase activity in ripe red fruit, together with an incomplete or non-cyclic operation of the tricarboxylic acid (TCA) cycle, as reflected by the higher citrate and malate contents. In the partial TCA cycle, one branch produces citrate while the other synthesizes malate (Igamberdiev and Eprintsev, 2016). The metabolite composition of fruit influences its flavor, which is determined by several factors including the sugar/acid ratio as an important determinant of taste (Zanor et al., 2009). Then, the metabolomics approach suggests a tastier tomato.

It is remarkable that fruit yield was maintained and sugar contents increased in plants expressing chloroplast Fld despite significant mass decreases in source tissue. Higher photosynthetic activity per leaf area (**Figure 3**) might contribute to this phenotype by providing higher source strength. While the possibility that the plastid-targeted flavoprotein affected source-sink assimilate partitioning cannot be ruled out, genes differentially expressed by chloroplast Fld in tobacco failed to reveal any obvious trait associated to this system (Bermúdez et al., 2008; Bermúdez et al., 2014; Pierella Karlusich et al., 2017).

Modification of HI as a key agronomical goal has been accomplished through various strategies. Most attempts have relied on crosses with wild relatives to select favorable alleles and on engineering hormone accumulation and signaling. Many introgression lines of *Solanum pennellii* did show high HI (Schauer et al., 2006), usually accompanied by decreased overall yields (Gur et al., 2010). On the other hand, brassinosteroid metabolism has been targeted to modify several traits in tomato including HI. Overexpression of the brassinosteroid receptor *SlBRI1* resulted in increased plant size and leaf area, with little or no effect on fruit yield (Nie et al., 2017). As a consequence, the overall HI was decreased in those plants, partly compensated by accelerated fruit ripening and improved quality. *DWARF*, the key brassinosteroid biosynthetic gene in tomato, has also been overexpressed, leading to significant increases in plant height and biomass, and lower expansion diameter (Li et al., 2016). The major decline in HI was partially compensated by early flowering and accelerated fruit ripening, and the authors predicted higher yield per planted surface due to the compact architecture of *DWARF*-expressing plants (Li et al., 2016).

In a different approach, improvement of HI was obtained by silencing the expression of a chloroplast DnaJ chaperone involved in assimilate partitioning into fruit (Bermúdez et al., 2014). The increase in HI was gained through higher ripe fruit weight per plant without modification of the aerial biomass (Bermúdez et al., 2014). Under the conditions employed in that trial, the HI of WT control plants was very low (∼0.1), compared to 0.48 in our assay (**Figure 4E**). Despite the more stringent background, *Slpfld* plants HI did increase to ∼0.63, even higher than those reported for DnaJ-silenced plants that were close to 0.5 (Bermúdez et al., 2014). Moreover, calculations based on the horizontal expansion diameters of the *Slpfld* plants (**Supplementary Figure S3B**), as done by Li et al. (2016), suggest that major improvements in absolute fruit yield per planted surface could be gained by increasing plant density per square meter in the field.

Tomato plants did not represent the only reported case of leaf size decrease upon expression of a plastid-located Fld. As indicated, creeping bentgrass (Li et al., 2017) and *Arabidopsis* (Su et al., 2018) displayed a similar phenotype. Interestingly, development of vegetative tissues in Fld-expressing tobacco (Ceccoli et al., 2012) and *Medicago truncaluta* (Coba de la Peña et al., 2010) lines did not differ significantly from those of their WT siblings, suggesting that the effect of the flavoprotein on growth might have some degree of species specificity. Cell size, however, was actually reduced in leaves of *pfld* tobacco plants (Mayta et al., 2018), prompting for a more detailed study of the developmental features displayed by these plants in the absence of stress.

The mechanisms by which chloroplast-targeted Fld can modulate organ development are presently unknown. Su et al. (2018) proposed that size reduction might reflect the lower efficiency of Fld, compared to Fd, as electron carrier during photosynthesis (Nogués et al., 2004). Alternatively, Fld could modulate redox processes and down-regulate accumulation of reactive oxygen species (ROS), as observed in Fld-expressing plants exposed to adverse environmental situations (Tognetti et al., 2006, Tognetti et al., 2007; Zurbriggen et al., 2008; Zurbriggen et al., 2009; Li et al., 2017; Rossi et al., 2017). Oxidative bursts have been detected during both leaf and fruit transitions (Muñoz and Munné-Bosch, 2018), and proposed to provide signaling cues required for these developmental programs. The cellular origin(s) of the observed ROS build-up are still unclear but they most likely involve chloroplasts and mitochondria (Muñoz and Munné-Bosch, 2018).

Ascorbate is a canonical plant antioxidant, and manipulation of its metabolism has been used to modify yield and HI in cherry tomatoes. The levels of ascorbate oxidase, which oxidizes ascorbate to monodehydroascorbate, and of monodehydroascorbate reductase (MDHAR), which participates in ascorbate regeneration, were reduced using RNAi techniques (Garchery et al., 2013; Truffault et al., 2016). Knocked-down ascorbate oxidase plants showed improved yield (Garchery et al., 2013), while the opposite effect was observed in siblings with impaired MDHAR activity (Truffault et al., 2016). The results revealed a strong correlation between antioxidant levels (in this case, ascorbate) and yield. Fruit-specific decreases of ascorbate oxidase and MDHAR activities obtained by expressing the RNAi sequences under control of a fruit promoter had no consequences in yield, indicating that the antioxidant capacity of leaves was the key factor determining the yield phenotype (Truffault et al., 2018). Moreover, application of this same strategy to Moneymaker tomatoes failed to show any fruit change (Truffault et al., 2018), underscoring the importance of the genotype in the determination of yield and HI. Cherry tomatoes produce small fruit and exhibit low HI values compared to Moneymaker, indicating that source-sink relationships and metabolite allocation must be necessarily different in the two cultivars.

MDHAR isoforms are distributed in various cellular compartments, whereas ascorbate oxidase is an apoplastic enzyme. Then, to the best of our knowledge this is the first report in which genetic manipulations of a chloroplast redox shuttle modify growth of a sink tissue and HI. The connection between plastid function and organ development has been recognized only recently (Andriankaja et al., 2012; Van Dingenen et al., 2016). Chloroplasts communicate information to nuclei as a response to environmental and developmental stimuli, a process known as retrograde signaling (Hernández-Verdeja and Strand, 2018). Several potential operating signals originating from these organelles have been proposed, including ROS and the redox status of the PETC and the chloroplast stroma (Exposito-Rodriguez et al., 2017), all of which can be affected by Fld presence (Pierella Karlusich et al., 2014; Rossi et al., 2017; Mayta et al., 2018). Organ size, on the other hand, has been related to various cellular processes including endoreduplication (Kawade and Sukaya, 2017), phytochromes (Husaineid et al., 2007), and modulation *via* proteasomal activity (Sonoda et al., 2009; Nguyen et al., 2013). Noteworthy, the ubiquitin-proteasome system has been reported to modulate plastid-nuclear bidirectional communication (Hirosawa et al., 2017), and most proteasomal components were up-regulated by chloroplast Fld presence in tobacco plants grown under normal conditions (Pierella Karlusich et al, 2017), suggesting that the effects of the flavoprotein could be mediated by selective protein degradation. Then, our working hypothesis is that, by productively interacting with the PETC and other oxido-reductive pathways of the chloroplast, Fld affects retrograde signaling involved in organ development, presumably mediated by proteasomal function, ploidy, receptors, etc. Research is currently underway to address these possibilities.

## Data Availability Statement

The datasets generated for this study are available on request to the corresponding author.

## Author Contributions

MM, MZ, EV, M-RH, MIZ, and NC conceived the original research plans. MM, RA, MZ, EV, M-RH, MIZ, and NC designed the experiments. MM, RA, MZ, EV, M-RH, and MIZ performed the experiments. MM, RA, MZ, EV, M-RH, MIZ, and NC analyzed the data. MM, RA, MZ, EV, M-RH, MIZ, and NC wrote the manuscript.

## Funding

This work was supported by grants PICT-2015-3828 and PICT-2017-1301 from the National Agency for the Promotion of Science and Technology (ANPCyT, Argentina). MM and RA are post-doctoral and doctoral Fellows, respectively, from the National Research Council (CONICET, Argentina). EV, MIZ, and NC are Staff Researchers from CONICET. MM, EV, MIZ, and NC are Faculty members of the School of Biochemical and Pharmaceutical Sciences, University of Rosario (Facultad de Ciencias Bioquímicas y Farmacéuticas, Universidad Nacional de Rosario, Argentina). MZ is a Faculty member of the Düsseldorf University.

## Conflict of Interest

The authors declare that the research was conducted in the absence of any commercial or financial relationships that could be construed as a potential conflict of interest.

## Abbreviations

Chl, chlorophyll; PS, photosystem; dpg, days post-germination; Fd, ferredoxin; Fld, flavodoxin; HI, harvest index; LEF, linear electron flow; MDHAR, monodehydroascorbate reductase; ^1^H-NMR, proton nuclear magnetic resonance; NPQt, non-photochemical quenching; PETC, photosynthetic electron transport chain; ROS, reactive oxygen species; SDS-PAGE, sodium dodecyl sulfate–polyacrylamide gel electrophoresis; TCA, tricarboxylic acid, TSP, 3-(trimethylsilyl) propionic-2,2,3,3-d4 acid; WT, wild-type.
