# Supplementary material for: Expression of a Chloroplast-Targeted Cyanobacterial Flavodoxin in Tomato Plants Increases Harvest Index by Altering Plant Size and Productivity
Source: Front Plant Sci. 2019 Nov 8;10:1432. doi: 10.3389/fpls.2019.01432 (PMC6865847; doi:10.3389/fpls.2019.01432)
Supplement: Supplementary file 1 [file DataSheet_1.pdf]

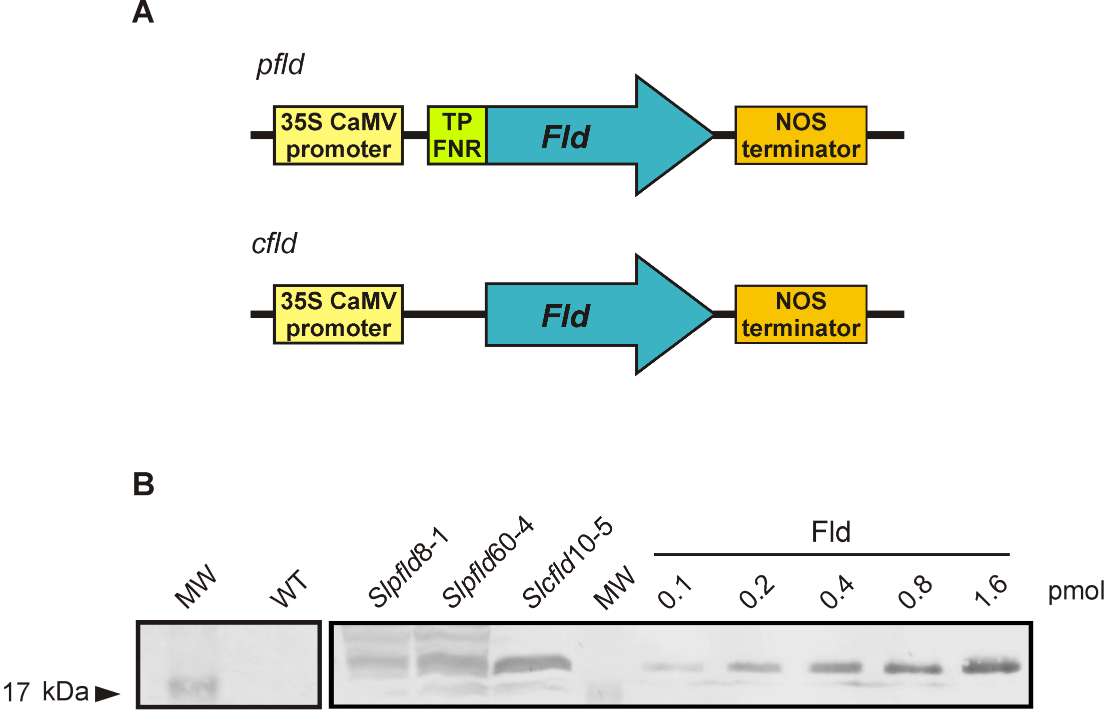

Supplementary Figure S1. Schematic diagrams of the T-DNA regions from vectors used for transformation. (A) Fld was translationally fused (*pfld*) or not (*cfld*) to the transit peptide of pea ferredoxin-NADP$^+$ reductase (TP FNR) for plastid or cytosol targeting, respectively. Both constructs were placed between the constitutive 35S promoter of the cauliflower mosaic virus (CaMV) and the transcriptional terminator of the nopaline synthase (NOS terminator). (B) Fld expression in leaf tissue from *Slpfld* and *Slcfld* lines as determined by SDS-PAGE and inmunoblot using Fld antisera (see Materials and Methods). The third leaflets of the fourth fully expanded leaves (counting from the bottom) of plants at 30 dpg were used to prepare the extracts. Soluble fractions corresponding to 5 mg FW were loaded in the lanes labeled WT, *Slpfld*8-1, *Slpfld*60-4 and *Slcfld*10-5. MW shows a molecular weight standard of 17 kDa. Lanes on the right correspond to different amounts (0.1-1.6 pmol) of purified recombinant Fld.
