# Supplementary material for: Expression of a Chloroplast-Targeted Cyanobacterial Flavodoxin in Tomato Plants Increases Harvest Index by Altering Plant Size and Productivity
Source: Front Plant Sci. 2019 Nov 8;10:1432. doi: 10.3389/fpls.2019.01432 (PMC6865847; doi:10.3389/fpls.2019.01432)
Supplement: Supplementary file 2 [file DataSheet_2.pdf]

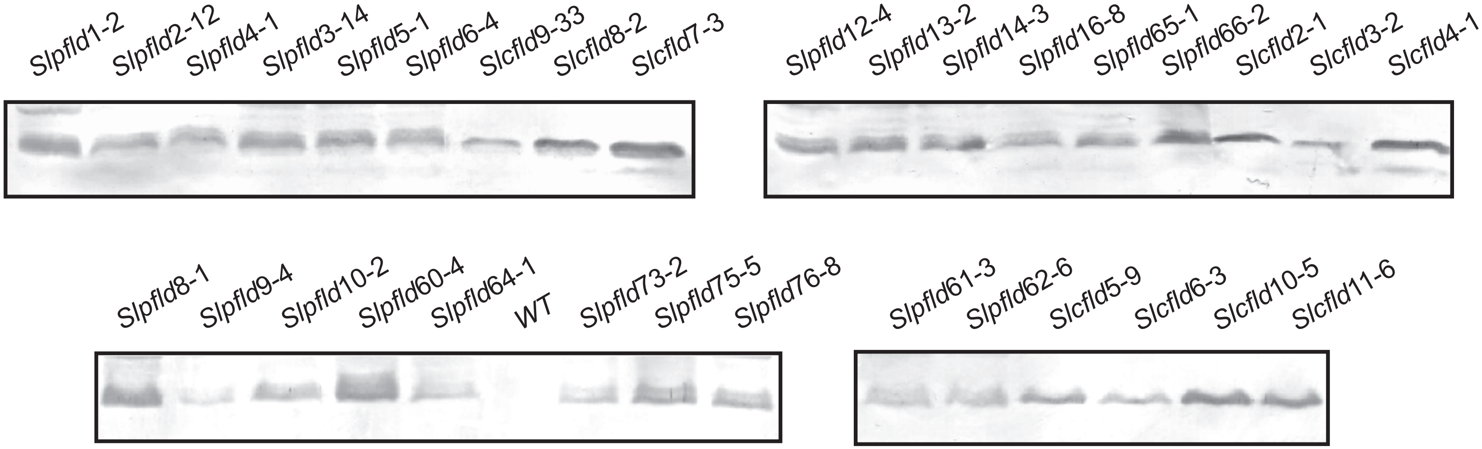

Supplementary Figure S2. Fld expression in leaf tissue from *Slpfld* and *Slcfld* transformants. Cleared extracts corresponding to 5 mg FW were loaded in each lane, resolved by 15% SDS-PAGE and analyzed by immunoblot using Fld antisera, as described in Materials and Methods. Lines are indicated above each lane.
