# Supplementary material for: Expression of a Chloroplast-Targeted Cyanobacterial Flavodoxin in Tomato Plants Increases Harvest Index by Altering Plant Size and Productivity
Source: Front Plant Sci. 2019 Nov 8;10:1432. doi: 10.3389/fpls.2019.01432 (PMC6865847; doi:10.3389/fpls.2019.01432)
Supplement: Supplementary file 3 [file DataSheet_3.pdf]

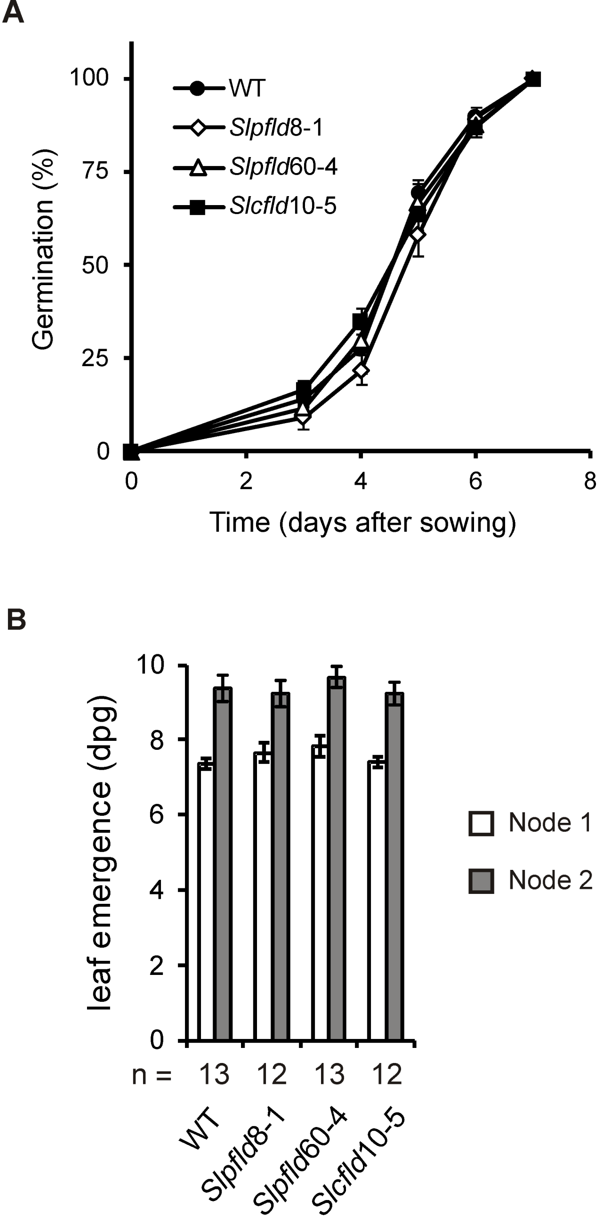

Supplementary Figure S3. Plastid-targeted Fld did not affect seed viability, germination rates or timing of leaf emergence. For determination of seed viability and germination rates (A), lots of 30 seeds from each genotype were cultivated in half-strength Murashige & Skoog medium under growth chamber conditions (see Materials and Methods). Data shown corresponds to means ± SEM of 4 independent experiments. Determination of time to leaf emergence (B) was performed using 12-13 plants germinated at the same time in soil as described in Materials and Methods. Data shown are means ± SEM.. No significant differences were found at $P < 0.05$ using ANOVA.
