# Supplementary material for: Expression of a Chloroplast-Targeted Cyanobacterial Flavodoxin in Tomato Plants Increases Harvest Index by Altering Plant Size and Productivity
Source: Front Plant Sci. 2019 Nov 8;10:1432. doi: 10.3389/fpls.2019.01432 (PMC6865847; doi:10.3389/fpls.2019.01432)
Supplement: Supplementary file 4 [file DataSheet_4.pdf]

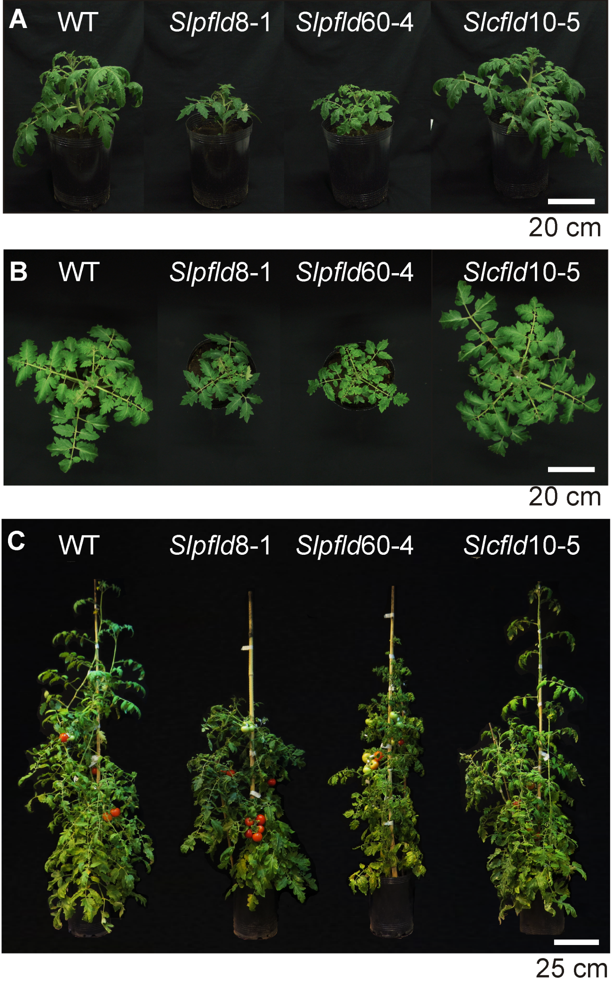

Supplementary Figure S4. Fld expression in tomato chloroplasts affected vegetative growth. Side (A) and top (B) views of plants at 35 dpg. (C) Phenotypes of plants at 116 dpg.
