# Supplementary material for: Expression of a Chloroplast-Targeted Cyanobacterial Flavodoxin in Tomato Plants Increases Harvest Index by Altering Plant Size and Productivity
Source: Front Plant Sci. 2019 Nov 8;10:1432. doi: 10.3389/fpls.2019.01432 (PMC6865847; doi:10.3389/fpls.2019.01432)
Supplement: Supplementary file 5 [file DataSheet_5.pdf]

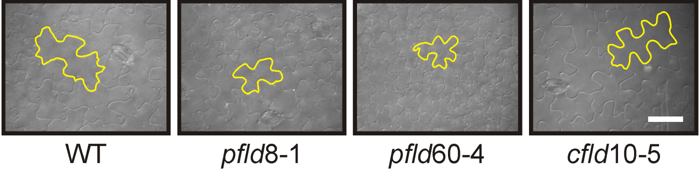

Supplementary Figure S5. Chloroplast Fld decreased leaf epidermal cell size. Representative epidermal cells are contoured in yellow. Bar = 20 μm. Sampling and microscopic analysis of leaf tissue were carried out as described in Materials and Methods.
