# Supplementary material for: Expression of a Chloroplast-Targeted Cyanobacterial Flavodoxin in Tomato Plants Increases Harvest Index by Altering Plant Size and Productivity
Source: Front Plant Sci. 2019 Nov 8;10:1432. doi: 10.3389/fpls.2019.01432 (PMC6865847; doi:10.3389/fpls.2019.01432)
Supplement: Supplementary file 6 [file DataSheet_6.pdf]

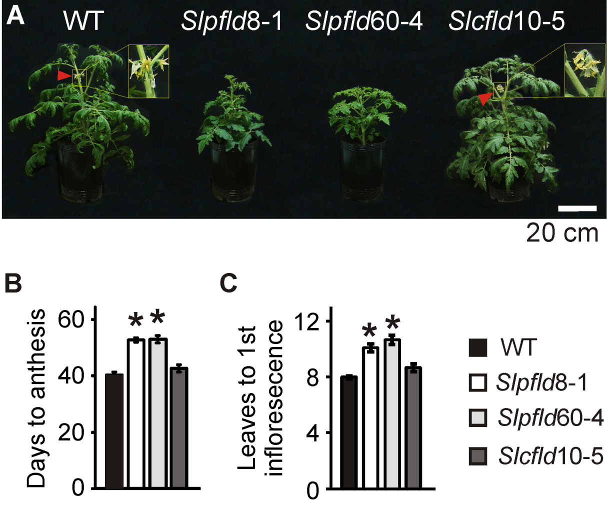

Supplementary Figure S6. Fld expression in chloroplasts delayed flowering in tomato. (A) Phenotypes of plants at 45 dpg. Insets show first flowers in WT and *Slcfld*10-5 plants. (B) Number of days to the first flower in anthesis. (C) Number of leaves at the appearance of the first inflorescence. Data reported are means ± SEM of 10-12 biological replicates. Asterisks indicate statistically significant differences and were determined using one-way ANOVA and Tukey's Multiple Comparison Test ($P < 0.05$).
