# Supplementary material for: Expression of a Chloroplast-Targeted Cyanobacterial Flavodoxin in Tomato Plants Increases Harvest Index by Altering Plant Size and Productivity
Source: Front Plant Sci. 2019 Nov 8;10:1432. doi: 10.3389/fpls.2019.01432 (PMC6865847; doi:10.3389/fpls.2019.01432)
Supplement: Supplementary file 7 [file DataSheet_7.pdf]

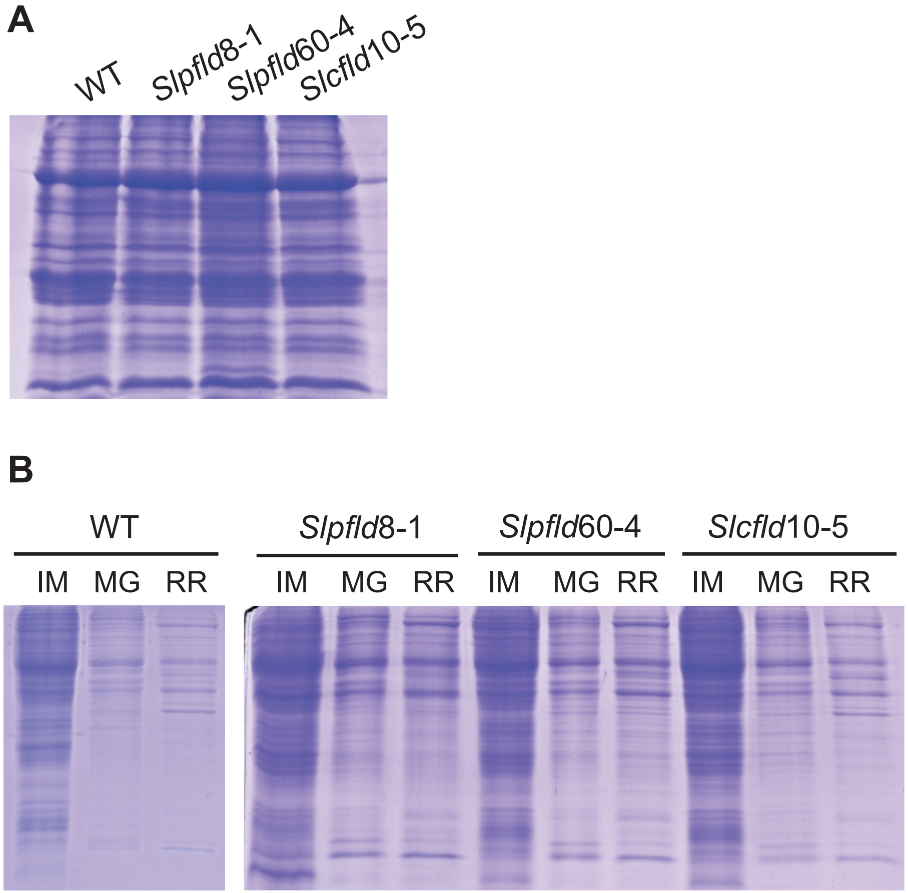


**Supplementary Figure S7.** Protein patterns of tomato leaves and fruit. Coomassie Brilliant Blue stain of a 15% SDS-PAGE of leaf (A) and fruit (B) extracts. Lysates corresponding to 5 mg FW of the various tissues were loaded in each lane.
