# Supplementary material for: Expression of a Chloroplast-Targeted Cyanobacterial Flavodoxin in Tomato Plants Increases Harvest Index by Altering Plant Size and Productivity
Source: Front Plant Sci. 2019 Nov 8;10:1432. doi: 10.3389/fpls.2019.01432 (PMC6865847; doi:10.3389/fpls.2019.01432)
Supplement: Supplementary file 8 [file DataSheet_8.pdf]

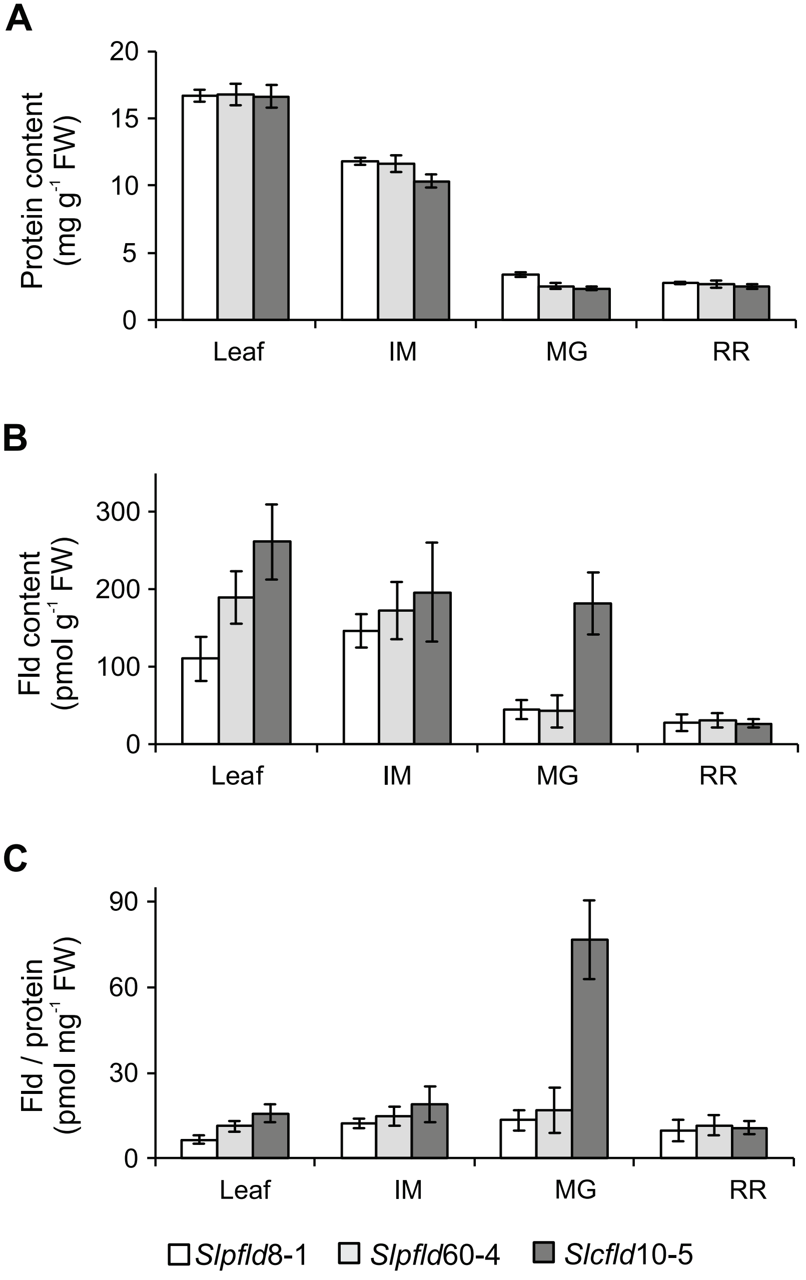

Supplementary Figure S8. Fld and protein levels declined during tomato fruit ripening. Total protein (A) and Fld (B) contents in cleared extracts from tomato leaf and fruit tissue. Values are means SEM (n = 3). Fld levels were estimated from immunoblots, by comparison with different amounts of purified recombinant Fld as illustrated in Supplementary Figure S1B. (C) Ratios of Fld to total protein were calculated from data of panels A and B for leaf and fruit tissue of *Slpfld* and *Slcfld* plants.
