# Supplementary material for: Expression of a Chloroplast-Targeted Cyanobacterial Flavodoxin in Tomato Plants Increases Harvest Index by Altering Plant Size and Productivity
Source: Front Plant Sci. 2019 Nov 8;10:1432. doi: 10.3389/fpls.2019.01432 (PMC6865847; doi:10.3389/fpls.2019.01432)
Supplement: Supplementary file 9 [file DataSheet_9.pdf]

Supplementary Table S1. Vegetative phenotypes of WT and Fld-expressing transgenic tomato lines.
Data are means ± SEM of n plants as indicated in parentheses. Statistically significant differences between Fld-expressing and WT plants are shown in bold and were determined using one-way ANOVA and Tukey's Multiple Comparison Test (P < 0.05).

Line	Average leaves per simpodial shoot[a]	Internode length[b] (cm)	Rachis length (cm)	Stem diameter[c] (cm)	FW aerial part[d] (g per plant)	DW aerial part[d] (g per plant)	Aerial part H_2O content[e] (%)
WT	3.0 ± 0.3 (12)	2.6 ± 0.1 (12)	17.9 ± 0.3 (8)	1.15 ± 0.03 (12)	153 ± 5 (18)	23.9 ± 3.2 (6)	84.2 ± 1.6 (6)
Slpfld 8-1	2.9 ± 0.1 (12)	**1.8 ± 0.1** (12)	**10.9 ± 0.3** (10)	1.06 ± 0.04 (12)	**118 ± 6** (19)	16.5 ± 1.1 (6)	83.7 ± 0.8 (6)
Slpfld 60-4	2.8 ± 0.2 (12)	**1.8 ± 0.1** (12)	**10.3 ± 0.6** (9)	1.18 ± 0.03 (12)	**119 ± 5** (20)	**15.0 ± 0.9** (6)	84.5 ± 0.6 (6)
Sl*cfld* 10-5	2.7 ± 0.2 (12)	2.3 ± 0.1 (12)	14.9 ± 1.0 (4)	1.20 ± 0.03 (12)	159 ± 13 (11)	18.9 ± 1.9 (6)	83.2 ± 1.3 (6)

[a] Number of leaves between 5 consecutive sympodia.
[b] Mean internode length between cotyledons and the first fully expanded leaf.
[c] Stem diameter corresponds to that of the fifth internode.
[d] Weight of aerial part (leaves+stem) corresponds to plants after fruit harvest.
[e] Water content calculated as ((FW - DW)/FW) x 100.

Supplementary Table S2. Reproductive phenotypes of WT and Fld-expressing transgenic tomato lines.
Data are means ± SEM of n plants as indicated in parentheses. Statistically significant differences between Fld-expressing and WT plants are shown in bold and were determined using one-way ANOVA and Tukey's Multiple Comparison Test (P < 0.05).

Line	Inflorescences per plant[a]	Flowers per inflorescence	Fruits per plant	Fruit production (g per plant)	Days to color break[b]	Days to fruit ripening[b]	Fruit FW (g per fruit)[c]	Pericarp DW (g per fruit)[d]	Pericarp H_2O content[e] (%)
WT	13.7 ± 1.7 (6)	5.9 ± 0.1 (12)	5.2 ± 0.4 (18)	149 ± 15 (18)	55.5 ± 1.0 (12)	63.8 ± 1.1 (12)	34 ± 3 (18)	2.65 ± 0.11 (11)	93.2 ± 0.3 (11)
Slpfld 8-1	12.5 ± 0.7 (6)	**8.9 ± 0.3** (12)	**8.0 ± 0.5** (20)	173 ± 15 (20)	**51.0 ± 1.0** (12)	**61.4 ± 0.8** (12)	**25 ± 1** (20)	2.01 ± 0.12 (11)	92.4 ± 0.5 (11)
Slpfld 60-4	15.3 ± 0.8 (6)	**8.8 ± 0.3** (12)	**8.6 ± 0.7** (20)	190 ± 16 (20)	**50.2 ± 0.8** (12)	**58.7 ± 0.8** (12)	**26 ± 2** (20)	2.05 ± 0.14 (11)	92.9 ± 0.4 (12)
Sl*cfld* 10-5	13.7 ± 1.4 (6)	6.3 ± 0.3 (12)	5.6 ± 0.6 (14)	155 ± 14 (15)	57.5 ± 0.8 (12)	68.9 ± 1.0 (12)	35 ± 3 (14)	2.31 ± 0.16 (11)	93.3 ± 0.3 (11)

[a] Total number of inflorescences (primary and secondary).
[b] Days to color break and ripening are counted from the anthesis of individual flowers (*n* >50).
[c] Calculated using all the red ripe fruits from each plant (*n* >4).
[d] Calculated using all the red ripe fruits from each plant (n>4) after removal of pulp and seeds.
[e] Water content calculated as ((FW - DW)/FW) x 100.

Supplementary Table S3. Metabolite levels in ripe red fruits of WT, *Slpfld* and *Slcfld* plants. Extracts were prepared from the first two ripe red fruit from at least 3 different plants of every line, and metabolite contents were determined as described in Materials and Methods. Values for each compound are expressed in µmol g^{-1} FW and are presented as means ± SEM. Evaluation of the statistical significance of differences for metabolite contents was performed using ANOVA followed by Fisher´s multiple comparison tests. Means with the same letter are not significantly different from each other at $P< 0.05$.

Metabolite level (µmol g^{-1} FW)	WT	*Slpfld*8-1	*Slpfld*60-4	*Slcfld*10-5
Sucrose	3.68 ± 1.0[b]	1.67 ± 0.3[ab]	1.06 ± 0.3[a]	2.77 ± 0.7[b]
Fructose	412.02 ± 15.5[a]	645.11 ± 37.3[c]	623.59 ± 82.7[bc]	453.61 ± 85.5[ab]
Glucose	521.46 ± 23.5[a]	813.22 ± 48.6[b]	693.83 ± 87.4[ab]	552.80 ± 117.2[a]
Galactose	19.71 ± 2.0[a]	24.03 ± 6.8[a]	22.67 ± 6.2[a]	17.37 ± 3.2[a]
Xylose	3.39 ± 2.0[a]	3.88 ± 0.9[a]	4.37 ± 2.1[a]	5.34 ± 2.3[a]
Mannose	18.58 ± 0.7[a]	23.25 ± 3.3[a]	20.67 ± 3.3[a]	16.74 ± 1.7[a]
Citrate	55.47 ± 1.7[a]	116.09 ± 5.7[b]	100.24 ± 28.8[ab]	54.51 ± 10.2[a]
Malate	57.89 ± 3.8[a]	116.62 ± 21.8[b]	99.62 ± 21.7[ab]	79.79 ± 19.9[ab]
Fumarate	2.30 ± 0.5[a]	3.75 ± 0.6[a]	2.19 ± 0.2[a]	2.39 ± 0.5[a]
Succinate	8.41 ± 0.2[ab]	16.64 ± 3.7[b]	15.21 ± 4.4[ab]	7.76 ± 1.7[a]
Pyruvate	16.66 ± 0.3[a]	35.78 ± 6.8[b]	31.24 ± 8.9[ab]	16.78 ± 2.6[a]
α-ketoglutarate	13.46 ± 1.7[a]	15.20 ± 0.8[a]	20.49 ± 9.5[a]	14.65 ± 3.1[a]
γ-aminobutyrate	15.03 ± 2.1[a]	36.96 ± 0.8[b]	27.34 ± 7.9[ab]	21.04 ± 5.9[ab]
Alanine	1.11 ± 0.0[a]	1.59 ± 0.2[a]	1.48 ± 0.4[a]	1.03 ± 0.0[a]
Asparagine	5.10 ± 0.18[a]	4.95 ± 0.8[a]	4.99 ± 1.8[a]	7.90 ± 3.9[a]
Aspartate	18.09 ± 5.4[a]	27.21 ± 12.2[a]	35.58 ± 26.7[a]	13.49 ± 2.6[a]
Glutamate	22.15 ± 0.6[a]	29.69 ± 2.9[a]	37.43 ± 17.6[a]	24.65 ± 4.7[a]
Glutamine	23.31 ± 0.7[a]	24.31 ± 1.0[a]	28.82 ± 12.4[a]	19.38 ± 3.0[a]
Isoleucine	0.80 ± 0.1[a]	0.99 ± 0.1[a]	1.05 ± 0.3[a]	0.72 ± 0.1[a]
Tyrosine	9.46 ± 1.0[a]	9.08 ± 0.1[a]	8.42 ± 2.2[a]	7.08 ± 1.4[a]
Phenylalanine	1.67 ± 0.2[a]	2.99 ± 0.6[a]	2.49 ± 0.7[a]	2.48 ± 1.2[a]
Threonine	1.27 ± 0.1[a]	1.44 ± 0.1[a]	1.49 ± 0.4[a]	1.53 ± 0.3[a]
Tryptophan	0.60 ± 0.1[a]	0.73 ± 0.1[a]	0.71 ± 0.3[a]	0.46 ± 0.1[a]
Valine	0.56 ± 0.0[a]	0.62 ± 0.1[a]	0.67 ± 0.2[a]	0.54 ± 0.1[a]
Trans-cinnamate	5.78 ± 0.8[a]	17.35 ±2.5[b]	6.24 ± 1.7[a]	5.90 ± 0.4[a]
